# Supplementary figures and images for: Study of Metakaolinite Geopolymeric Mortar with Plastic Waste Replacing the Sand: Effects on the Mechanical Properties, Microstructure, and Efflorescence
Source: Materials (Basel). 2022 Dec 2;15(23):8626. doi: 10.3390/ma15238626 (PMC9737107; doi:10.3390/ma15238626)

## Supplementary Materials

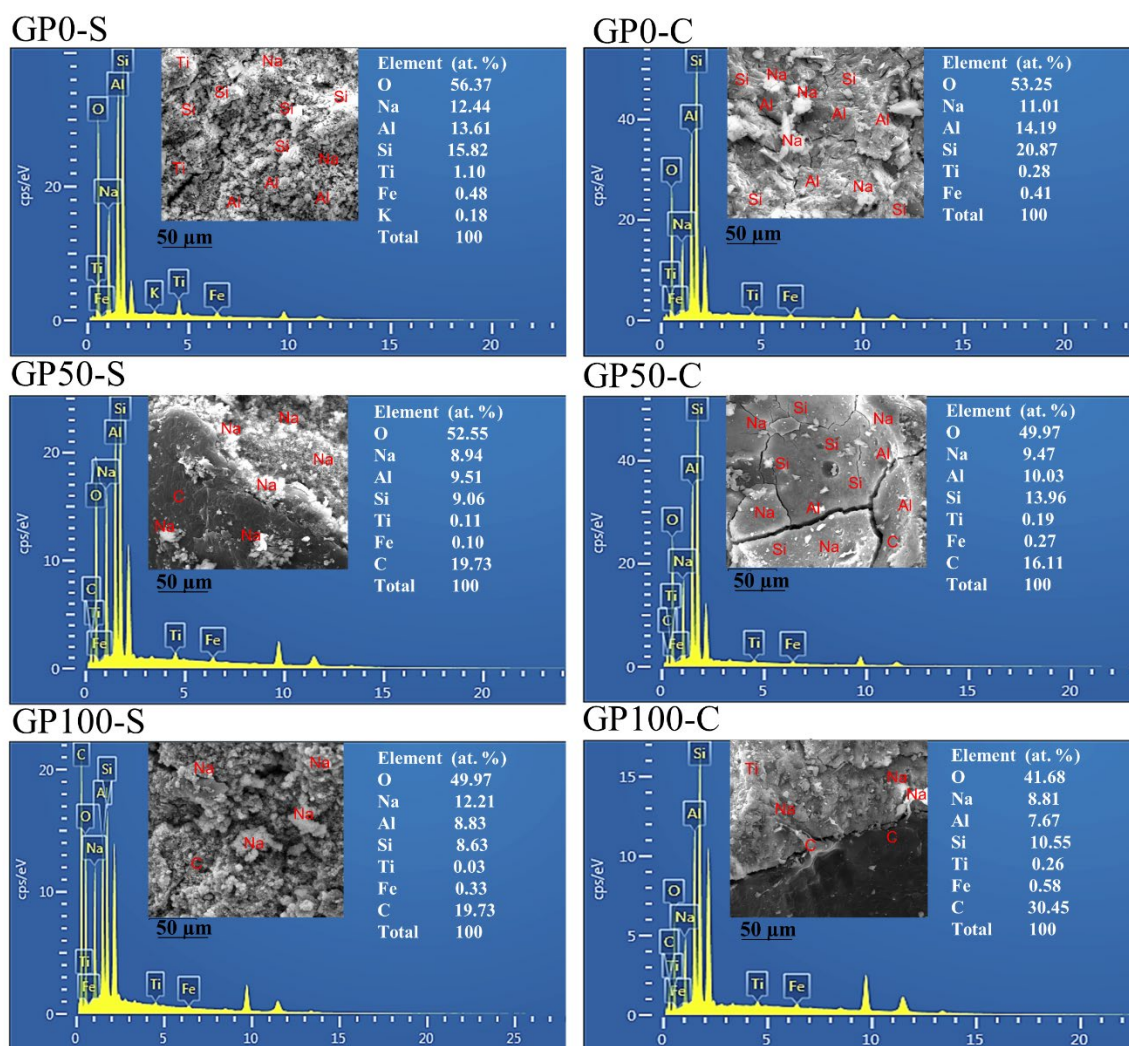

**Figure S1:** EDS of the mortars of the groups GP-S and GP-C.

Supplement: Supplementary file 1 [file materials-15-08626-s001.zip › materials-1962948-supplementary.pdf]
